# Risk factors associated with retinopathy in young people with type 1 diabetes in Bangladesh

**DOI:** 10.1002/edm2.197

**Published:** 2020-12-17

**Authors:** Bedowra Zabeen, Mohammad Zafar Khaled, Lutful Husain, Asma Aktar, Kamrul Huda, Yeasmin Afroz Kamal, Nujhat Choudhury, Kishwar Azad

**Affiliations:** ^1^ Department of Paediatrics Life for a Child & Changing Diabetes in Children Programme Bangladesh Institute of Research & Rehabilitation in Diabetes, Endocrine & Metabolic Disorders (BIRDEM) Diabetic Association of Bangladesh Dhaka Bangladesh; ^2^ Department of Ophthalmology Bangobandhu Sheikh Mujib Medical University (BSMMU) Dhaka Bangladesh; ^3^ Orbis International Bangladesh Country Office Dhaka Bangladesh; ^4^ National Institute of Ophthalmology (NIO) Dhaka Bangladesh; ^5^ Life for a child (LFAC) and Changing Diabetes in Children (CDiC) Programme BIRDEM 2 Diabetic Association of Bangladesh Dhaka Bangladesh; ^6^ Department of Ophthalmology Bangladesh Institute of Research & Rehabilitation in Diabetes, Endocrine & Metabolic Disorders (BIRDEM) Dhaka Bangladesh; ^7^ Department of Paediatrics Perinatal Care Project Bangladesh Institute of Research & Rehabilitation in Diabetes, Endocrine & Metabolic Disorders (BIRDEM) Diabetic Association of Bangladesh Dhaka Bangladesh

**Keywords:** Bangladesh, retinopathy, risk factors, type 1 diabetes

## Abstract

**Objectives:**

Diabetic retinopathy (DR) is the most common microvascular complications seen in children and adolescents with type 1 diabetes. The aim of this study was to evaluate the prevalence of retinopathy and its association with other risk factors in young people with type 1 diabetes.

**Methods:**

This study was a cross‐sectional study, which was done as part of the ongoing complication assessment in the paediatric diabetes clinic in BIRDEM (Bangladesh Institute of Research and Rehabilitation of Diabetes Endocrine and Metabolic Disorders), a tertiary care hospital. Children, adolescents and young adults with type 1 diabetes who were having diabetes duration >2 years were included in this study. Retinopathy was detected using fundal photography, and grading was done by National Screening Committee of UK by trained ophthalmologists.

**Results:**

Diabetic retinopathy was observed in 44 (6.6%) patients. Majority (95.4%) of them had early diabetic retinopathy in the form of mild NPDR (nonproliferative diabetic retinopathy) (R1). Patients with retinopathy had higher HbA1c 9.6[8.4‐12.3] vs 9.1 [7.9‐10.8] (*P =* .013), longer duration of diabetes 7.6 [5.5‐10.7] vs 6.0 [4.5‐8.2] years (*P* = .001) and were older 21.5 [18.0‐23.0] vs 18 [16.0‐21.0] years (*P* = .0001) compared with those without retinopathy. On multivariate regression analysis, higher age and median HbA1c were significantly associated with DR.

**Conclusions:**

Higher HbA1c was the only modifiable risk factor for development of DR in our study population. Early detection of DR with improvement of glycaemic control may reduce the risk of progression of severe stages of the disease.

## Graphical abstract

The aim of this study was to investigate the prevalence and risk factors associated with, retinopathy in Youth with type 1 diabetes in Bangladesh. Diabetic retinopathy was observed in 44 (6.6%) patients. Majority 42 (95.4%) of them had early diabetic retinopathy mild NPDR (R1).

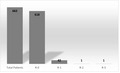

## 1 INTRODUCTION

Diabetic Retinopathy (DR) is the most common microvascular disease seen in children and adolescents with type 1 diabetes which is often asymptomatic in early stages but may progress to severe disease.^1^, ^2^, ^3^, ^4^, ^5^ The rising number of type 1 and type 2 diabetes in children has led to an increase number of young people at risk of visual problem.^6^, ^7^, ^8^ Almost all type 1 diabetes patients have developed some degree of retinopathy after 20 years of diabetes duration found in a study done by DCCT (Diabetes Control and Complications Trial) Research Group.^9^ The pathogenesis is still not clear; various factors—metabolic, environmental, hormonal and genetic factors, may play role in development of diabetic microangiopathy. The prevalence and risk factors of DR vary widely in different studies ranged from 0% to 28%.^10^, ^11^, ^12^, ^13^, ^14^ Although the prevalence of diabetic retinopathy in adolescents was quite high, approximately 41%‐42% in Australia and the United States and even higher in some European centres (46%)^15^, ^16^, ^17^ but recently lower prevalence rate (3.9%) has also been reported.^18^ Many studies reported that duration of 8‐10 years is required for the development of DR whereas few studies reported that mild DR occurred with short duration of DM (1‐2 years).^19^, ^20^, ^21^, ^22^ The risk factors for the development of DR include long duration of diabetes, poor glycaemic control, hypertension, hyperlipidaemia and genetic predisposition.^23^, ^24^, ^25^ The importance of strict glycaemic control and blood pressure control has been shown to reduce the progression of DR in the Diabetes Control and Complication Trial.^9^ ISPAD (International Society for Paediatric and Adolescent Diabetes) recommends screening for diabetic retinopathy should start from age 11 years with 2‐5 years diabetes duration.^26^ Whereas ADA (American Diabetes Association) and AAO (American Academy of Ophthalmology) recommend eye examination once youth have had type 1 diabetes for 3*‐*5 years, provided they are of age ≥10 years or puberty has started, whichever is earlier.^27^

There has been only one study done on development of DR, and associated risk factors in young patients with T1DM in Bangladesh. Hence, the purpose of this study was to assess the prevalence of retinopathy in children, adolescents and young adults with type 1 diabetes and further explore the risk factors for the development of DR.

We evaluated the DR among young people in type 1 diabetes enrolled in a largely managed comprehensive diabetes care in CDiC (Changing Diabetes in Children) and LFAC (Life for a Child) Paediatric Diabetes Center in BIRDEM (Bangladesh Institute of Research and Rehabilitation of Diabetes Endocrine and Metabolic Disorders) hospital in Bangladesh. We specifically focused on the association between retinopathy and recognized risk factors—age, diabetes duration, insulin dose and HbA1c.

## 2 RESEARCH DESIGN AND METHODS

This study was a cross‐sectional study, undertaken as part of the ongoing complication assessment in the CDiC and LFAC Paediatric Diabetes Center in BIRDEM 2, a tertiary care hospital. We analysed comprehensive data from patients with type 1 diabetes over 1 year period from October 2016 to October 2017 who were screened for DR. Children, adolescents and young adults with type 1 diabetes who were having diabetes duration >2 years were included in this study. Our centre is the only multidisciplinary centre where patients are referred from all over Bangladesh. Determination of the type 1 diabetes was made by the local and ISPAD criteria according to available clinical features and history: T1D was diagnosed upon abrupt onset of typical symptoms of diabetes with insulin required from diagnosis and no sign of insulin resistance—acanthosis nigricans and usually nonobese.^28^, ^29^ Clinical assessment was done by paediatric endocrinologist and the team during complications assessment.

The protocol was approved by the ethics committee of Diabetic Association of Bangladesh.

### 2.1 Assessment of Diabetic retinopathy

Screening for retinopathy has been started since 2016 in our clinic. All patients who came for follow‐up were screened (while they were ≥10 years old).

Colour fundus photography (CFP) was done in both eyes by using canon CR‐2 digital retinal camera by optometrist. Screening and grading for the presence or absence of diabetic retinopathy were performed by trained ophthalmologists. Grading was done by Disease Grading Protocol in National Guidelines on Screening for Diabetic Retinopathy Grading in England and Wales screening programmes.^30^

Data were collected in predesigned data collection sheet. HbA1c (haemoglobin A1c) done during the screening was taken for analysis. The informed consent from patients and their caregivers was taken during complication assessment.

### 2.2 Statistical analysis

Summary statistics are reported as mean ± SD for normally distributed data and median (interquartile range, IQR) for skewed data. While comparing the data, parametric test—ANOVA test was used for continuous data and nonparametric test—Kruskal‐Wallis test was used for skewed data. A probability ‘*P*’ of equal or less than .05 was considered significant and value more than 0.05 was not significant.

Risk factors for the development of retinopathy were assessed using logistic regression. Potential variables used in the regression model were age during the examination, HbA1c and diabetes duration.

## 3 RESULTS

**FIGURE 1:**
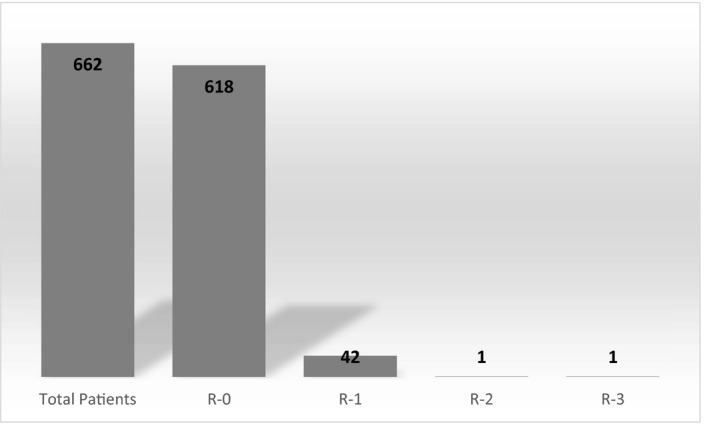
Grading of Retinopathy among the type 1 diabetes patients

Overall 622 patients were assessed who met the inclusion criteria. The median age of onset of diabetes was 12.0[9.0‐14.0] years in our study population. The median age during assessment was 19.0 [IQR: 16.0‐ 21.0] years and median duration of diabetes 6.1 [IQR: 4.5‐8.3] years. A total of 295 (44.6%) were male and 367(55.4%) were female. Diabetic retinopathy was observed in 44 (6.6%) patients. Majority (95.4%) of them had early diabetic retinopathy mild NPDR (R1), one patient had severe NPDR (R2), and one had proliferative retinopathy (R3) (Figure 1). The median diabetes duration was 7.6 [5.6‐10.7] years in patients with retinopathy. The earliest retinopathy developed in 4 years 5 months in one patient. While comparing the residence, the patients from rural had more retinopathy than urban area (*P *= .040) (Table 1). Patients with retinopathy had higher HbA1c 9.6[8.4‐12.3] vs 9.1 [7.9‐10.8] (*P* = ..013), longer duration of diabetes 7.6[5.6‐10.7] vs 6.0 [4.5‐8.2] years (*P* = .001) and were older 21.5 [18.0‐23.0] vs 18 [16.0‐21.0] years (*P* = .0001) compared to patients without retinopathy (Table 1).

**TABLE 1:** Characteristics of Patients with and without retinopathy

Parameters	Without retinopathy (n = 578)	With retinopathy (n = 44)	*P* value
Age at assessment	18.0 [16.0‐21.0]	21.5 [18.0‐23.0]	.0001
Age at diagnosis	12.0 [9.0‐14.0]	13.0 [10.0‐15.0]	.165
Sex			
Male	276 (93.6%)	19 (6.4%)	.849
Female	342 (93.2%)	25 (6.8%)	
Area of residence			
Urban	180 (97.3%)	5(2.7%)	.040
Semi‐urban	39(92.9%)	3 (7.1%)	
Rural	330 (91.7%)	30 (8.3%)	
Family history			
Yes	13 (5.7%)	13 (5.3%)	.954
No	214 (94.3%)	231 (94.7%)	
Diabetes duration (y)	6.0 [4.5‐8.2]	7.6 [5.6‐10.7]	.001
Insulin dose (U/d)	34 [26‐46]	36 [26‐46]	.447
HbA1c			
(%)	9.1 [7.9‐10.8]	9.6 [8.4‐12.3]	.013
mmol/mol	76.0 [62.8‐94.5]	81.4 [68.3‐110.9]	

### 3.1 Regression analysis of complication outcome

Retinopathy was associated with higher age, longer diabetes duration and higher HbA_1c_. Independent predictors of retinopathy were higher age, longer duration of diabetes and high HbA1c on univariate analysis. Higher age and HbA1c remained significantly associated with DR on multivariate analysis (Table 2).

**TABLE 2:** Factors associated with retinopathy in adolescents with diabetes

Factors	Univariate model		Multivariate model	
	OR [95% CI]	*P* value	OR [95% CI]	*P* value
Age at assessment	1.2 [1.09‐1.31]	.0001	1.16 [1.06‐1.27]	.001
Diabetes duration	1.15 [1.06‐1.25]	.001	1.08 [1.00‐1.19]	.082
HbA1c	1.18 [1.04‐1.34]	.008	1.15 [1.01‐1.30]	.031

Note

Age at diagnosis, sex and insulin dose were adjusted for the analysis.

## 4 DISCUSSION

The prevalence of retinopathy was found 6.6% in this cohort of 662 patients. In many studies, the prevalence of DR ranged from 0% to 28%.^10^, ^11^, ^12^, ^13^, ^14^ A large cohort study of 1604 patients in Australia, retinopathy was found in approximately 50% of adolescents with type 1 diabetes in the early years (1990s) which reduced to 12% (i2000s), which might be due to intensive management in recent years.^31^ In our study population, majority had early retinopathy R1 44 (95.4%). Similar finding (early retinopathy) was found in our previous study done in young patients with diabetes.^32^ Majority had early retinopathy in a study done in 119 children with type 1 diabetes in UK.^33^

Patients who are residing in rural area had more retinopathy than urban people in our study. It may be explained that the patients who are from rural area cannot visit the centre regularly and miss follow‐up often as they have to travel a long distance to come to the centre, which might also cause poorer metabolic control compared to urban people. In our study population, age of onset of diabetes was mostly at adolescence and higher age during assessment was conferred as a risk of DR. In a cohort of children with T1D in Australia, it was found that children who were diagnosed before the age of 5 years had a longer retinopathy free period than those diagnosed between 5 and 15 years.^34^ The increase incidence of DR during puberty probably due to hormonal influences which may affect the microvascular cellular integrity.^34^

Our patients who developed DR had longer median duration of diabetes (7.6 years) compared to non‐DR. In US SEARCH study of 265 young diabetics <20 years of age, 17% T1DM developed DR on fundus photography with median duration of 6.8 years.^35^ Conversely, a large cohort of 2240 youth, 20% of had developed diabetic retinopathy at a shorter duration (median duration of 3.2 years).^36^

Though there are several factors that may influence in development of DR but till now HbA1c has been found to be a modifiable risk factor. Higher HbA1c has been identified as a risk factor for not only in the development but also in progression of retinopathy in adolescents and adults.^37^, ^38^

Similar to other studies, we found the only modifiable risk factor was higher HbA1c for development of DR in our study population.^32^, ^39^, ^40^, ^41^, ^42^, ^43^, ^44^ Glycaemia showed a continuous relationship with retinopathy; therefore, emphasis should be given on strict glycaemic control in type 1 diabetes patient to reduce the risk of development and progression of DR. Screening for identification of early retinopathy with optimal glycaemic control should be part of management of type 1 diabetes.

On multivariate analysis, the risks of retinopathy were higher age and median HbA1c. Improved glycaemic control certainly reduces the risk of retinopathy which can be thought of as preventative strategies. Adolescents with poor glycaemic control have a higher risk of rapid progression to severe stages of retinopathy (severe nonproliferative retinopathy) as of adults.^45^, ^46^ Therefore, screening from adolescence time should be started for detection of early stage of diabetic retinopathy and emphasis on reduction of HbA1c should be given as improved glycaemic control may reduce or reverse the retinopathy.^47^, ^48^

The strength of this study included the sample size, with 662 young people with type 1 diabetes, representative of the different age groups from adolescents to young adults. This was an observational study and patient characteristics were comparable. It was a well‐described representative population, as patients were referred from almost all parts of the country to this centre. The data were collected during the complication assessment visit. All patients were assessed at single centre, and same protocol was used in the complications assessment methods throughout the study period, and the same trained ophthalmologists did the grading of the retinal images.

The limitations of the study were‐ this was a cross‐sectional study, thus was limited to observed associations and also not including all the modifiable risk factors which could reveal the other risk factors for prediction of retinopathy in addition to glycaemic control.

In conclusion, we found there is prevalence (6.6%) of retinopathy which was associated with higher age and HbA1c in young people with T1 D. Our results provide strong support for prior literature in emphasizing the early screening of DR and optimal glycaemic control to reduce the risk and progression of retinopathy. Longitudinal follow‐up of this cohort may provide the information about the predictive value of retinopathy and its further progression.

## CONFLICTS OF INTEREST

The authors declare that they have no potential conflicts of interest relevant to this article.

## AUTHOR'S CONTRIBUTION

BZ, ZK, NC and KA conceptualized and designed the study. BZ and ZK prepared the first draft of the manuscript. L H, K H, AA and Y K contributed to data preservation and analysis tools. All authors have contributed to manuscript revisions and read the manuscript. BZ, ZK and KA approved the final manuscript.
